# The application and perspective of hyperbaric oxygen therapy in acute ischemic stroke: From the bench to a starter?

**DOI:** 10.3389/fneur.2022.928802

**Published:** 2022-08-05

**Authors:** Yujia Yan, Xiqiang Zhang, Xingwei An, Wanpeng Fan, Jingbo Liang, Bin Luo, Hecheng Ren, Ying Huang

**Affiliations:** ^1^Academy of Medical Engineering and Translational Medicine, Tianjin University, Tianjin, China; ^2^Department of Neurosurgery, Tianjin Huanhu Hospital, Tianjin, China; ^3^Department of Neurosurgery, Third People's Hospital of Xining City, Xining, China; ^4^Tianjin Center for Brain Science, Tianjin, China

**Keywords:** stroke, acute ischemic stroke, hyperbaric oxygen therapy, neuroprotection, clinical application

## Abstract

Stroke has become a significant cause of death and disability globally. Along with the transition of the world's aging population, the incidence of acute ischemic stroke is increasing year by year. Even with effective treatment modalities, patients are not guaranteed to have a good prognosis. The treatment model combining intravenous thrombolysis/endovascular therapy and neuroprotection is gradually being recognized. After the clinical translation of pharmacological neuroprotective agents failed, non-pharmacological physical neuroprotective agents have rekindled hope. We performed a literature review using the National Center for Biotechnology Information (NCBI) PubMed database for studies that focused on the application of hyperbaric oxygen therapy in acute ischemic stroke. In this review, we present the history and mechanisms of hyperbaric oxygen therapy, focusing on the current status, outcomes, current challenges, perspective, safety, and complications of the application of hyperbaric oxygen in animal experiments and human clinical trials. Hyperbaric oxygen therapy, a non-pharmacological treatment, can improve the oxygenation level at the ischemic lesions in increased dissolved oxygen and oxygen diffusion radius to achieve salvage of neurological function, giving a new meaning to acute ischemic stroke.

## Introduction

Stroke is the second leading cause of death after coronary heart disease and the third leading cause of disability worldwide, according to the 2019 survey results. The number of new stroke cases and deaths increases each year substantially, and the patients are younger (1). The pathological subtypes include ischemic stroke (IS), intracerebral hemorrhage (ICH), and subarachnoid hemorrhage (SAH) (2). Ischemic strokes account for 62.4% of all the stroke events (1, 3, 4). The current treatment challenges are the high incidence, extremely high cost of treatment, and poor prognosis (5, 6).

The primary goal of acute ischemic stroke (AIS) treatment is revascularization and reperfusion of brain tissue (6, 7). However, despite effective intravenous thrombolysis (IVT) therapy and endovascular therapy (EVT) at an early stage, it does not mean that there is a good prognosis for the patient (8, 9). The medical fraternity has recognized the therapeutic model of recanalization and reperfusion combined with neuroprotection. However, all the 114 neuroprotective drugs currently in phase 2 and phase 3 clinical trials have failed in the clinical application; the single target of drug action and the necessity of the blood transport route have become important reasons for failure (10). Non-pharmacological neuroprotective measures such as hyperbaric oxygen therapy (HBOT) can exert neuroprotective effects through multiple pathways and targets, providing a new direction for neuroprotection treatment (11, 12). Therefore, this article mainly reviews the mechanism of HBOT, the current and controversial application in AIS, prospects, and analyzes the feasibility and safety of the application.

## Literature search and review criteria

This review follows the Preferred Reporting Items for Systematic Reviews and Meta-Analyses (PRISMA) statement (13). All the data are publicly available in the National Center for Biotechnology Information (NCBI) PubMed database. We screened publications in PubMed up to 31 March 2022 for full-text articles in English, using (“stroke” or “ischemic stroke” or “cerebral ischemia” or “cerebral ischemia stroke”) and (“hyperbaric oxygen” or “hyperbaric oxygen therapy” or “HBOT”).

The final selection of studies included an assessment of the effect of HBOT on AIS with the inclusion criteria of at least one of the following outcome measures: brain infarct size, neuronal survival, functional, safety and tolerability of intervention, and neurological outcomes, including but not limited to the modified Rankin Scale (mRS) at 90 days post-stroke. The exclusion criteria included non-English articles, AIS onset of more than 24 h, no reperfusion therapy or not reported, unclear time window for HBOT treatment or unclear indicators of total HBOT time, pressure and oxygen concentration, no control group in the trial, and <90 days of follow-up.

## History and mechanisms of hyperbaric oxygen therapy

Hyperbaric oxygen therapy is a non-pharmacological and non-invasive treatment for breathing with 100% oxygen at an ambient pressure >1.0 atmosphere absolute (ATA) (14). It has been widely used with satisfactory results in acute carbon monoxide and cyanide poisoning, gas gangrene, gas embolism, decompression sickness, and wound recovery (15, 16).

Before Priestley discovered the oxygen component of air, compressed air therapy, the predecessor of HBOT, was advocated as a medical treatment for certain acute and chronic diseases, but the original idea lacked a scientific basis (17). Later in 1879, French surgeon Fontaine established a mobile air-pressurized operating room to increase the effective oxygen content, making surgery with nitric oxide (NO) as the anesthetic safer (18). It was not until 1956, when Churchill–Davidson and Boerema conducted clinical trials of radiotherapy and cardiac surgery in a hyperbaric oxygen environment with satisfactory therapeutic results that the era of the scientific use of HBOT in modern medicine was marked (19, 20).

The most effective mechanism of action of hyperbaric oxygen is to increase the partial pressure of oxygen and the content of dissolved oxygen in the blood. Under normobaric conditions (1 ATA, 21% O_2_), the hemoglobin in the blood is already saturated (SaO_2_ ≈ 97%), and only 0.3 ml of oxygen per 100 ml of blood in the form of physically dissolved (21). High concentrations of oxygen and hyperbaric oxygen barely increase the amount of oxygen bound to hemoglobin anymore, but significantly increase the physical dissolved oxygen content in the blood. Based on the arterial-venous (A-V) O_2_ difference, 5% V/V of oxygen is offloaded to the tissue fluids. In the condition of 2.5 ATA, and 100% O_2_, 5.4% V/V of oxygen is physically dissolved in the plasma, which is theoretically sufficient to meet the basic physiological needs of the entire body (22).

The most famous is the “Life without blood” experiment conducted by Boerema in 1960 (23). The piglet maintained normal life activities after complete blood replacement in a hyperbaric environment (3 ATA, 100% O_2_), but died immediately after being removed from the hyperbaric environment. This experiment demonstrates that even in the absence of hemoglobin, the basic requirements for life survival can be maintained by relying on dissolved oxygen. In addition, the smaller molecular weight of oxygen compared to hemoglobin makes it easier to cross the blood–brain barrier, and the increase in dissolved oxygen reduces the dependence of brain tissue on hemoglobin-bound oxygen (24).

The mechanism of the effect of HBOT with multiple targets and pathways has been explained and demonstrated in detail in a large number of literature studies, so we will not elaborate on it in this article. The following beneficial effects of HBOT have been reported in AIS: (1) Reduce cerebrospinal fluid lactate and pyruvate levels and improve mitochondrial aerobic metabolism (25); (2) Reduce the area of ischemic focal ATP depletion and early tissue acidosis (26); (3) Regulate cerebrovascular flow, promote the formation of collateral circulation, and regulate driving blood into the ischemic region (25, 27); (4) Improve the effects of microcirculation and stabilizing the blood–brain barrier (28); and (5) Reduce the expression of hypoxia-inducible factor-1α (HIF-1α) and its downstream effector proteins and to inhibit apoptosis of neuronal cells and the inflammatory response (29, 30).

## Exploration of hyperbaric oxygen therapy in animal models

Hypoxic injury plays a central role in AIS. The impaired blood flow and oxygen supply to distal brain tissue due to arterial occlusion, and the high energy demand and low reserve capacity of neurons make cells hypoxic and rapidly lead to impaired oxidative metabolism in mitochondria. Dead neurons form the core of infarct foci and induce a multipathway molecular cascade of responses, including excitotoxicity, ion imbalance, oxidative and nitrosative reactions, inflammation, and apoptosis (31, 32). The area surrounding the core infarct, which is vulnerable to blood flow and metabolic changes, is called the “ischemic penumbra” region (16, 33). Salvaging the ischemic penumbra by IVT or EVT seems to lead to better clinical outcomes (8, 9, 34). PET studies have found that most patients have ischemic penumbra lasting up to 18 h, which provides a theoretical window for the effective treatment of AIS (33).

However, relying only on recanalization or reperfusion evaluation (modified Thrombolysis in Cerebral Infarction scale 2b or 3) makes the treatment decision for AIS too simple. Reperfusion does not imply a good prognosis (DAWN Trial Investigators 84 vs. 49%/DEFUSE 3 Investigators 76 vs. 45%), and the difference between them is not simply between the figures, which indicates that extra treatment strategies may be necessary. Pharmacological neuroprotection is limited by blood transport and a single action target, making the effect unsatisfactory (35). Thus, searching for non-pharmacological, physical neuroprotective treatment modalities may be a new approach.

Hyperbaric oxygen therapy can increase the dissolved oxygen content and increase the radius of oxygen dispersion to rapidly disperse oxygen to the ischemic site where the blood cannot reach. It is logical to perform HBOT in order to salvage the ischemic penumbra to avoid further increased infarct size and save neurological damage by increasing the oxygenation level of ischemic tissues. Experimental results in animal models of AIS suggest that the benefit of HBOT is mainly limited to the 12 h (time from the onset of AIS to the start of HBOT) (36). Most studies used a model of manual embolization of the middle cerebral artery in mice/rats. After temporary occlusion of the middle cerebral artery, HBOT within 0–8 h from onset significantly improves survival, reduces infarct volume, contributes to neurological recovery, and has a positive effect (Table 1). However, the benefits of HBOT are time-bound. In the study by Badr and Lou et al., it was found that HBOT performed for more than 12 and 23 h showed unfavorable outcomes with worse neurological function and larger infarct size (37, 38). Regretfully, there is no further explanation for this phenomenon, merely the suspicion that the administration of HBOT in severely ischemic tissues may lead to increased sensitivity of mitochondria to oxidative stress, resulting in more severe neurological impairment. In addition, the therapeutic effect of HBOT is also influenced by the recanalization after vascular occlusion. In animal models of permanent embolism, the time window of HBOT is even shorter. HBOT within 2 h showed a beneficial neuroprotective outcome, while treatment at 3/6 h had no significant effect (36, 39).

**Table 1:** Experimental results of animal models of temporary middle cerebral artery occlusion treated with HBOT.

**Authors**	**Animal**	**Ischemia conditions**	**Time window of HBOT**	**Dosing of HBOT**	**Effect**
Yu	Rats	MCA	0 min	2.0 ATA 100%O_2_ 1 h	Protective effects Reduce the infarct volume
Wang	Rats	MCA	0 min	2.5 ATA 100%O_2_ 1 h	Reduced the infarct volume Alleviated oxidative stress
Sunami	Rats	MCA	10 min	3.0 ATA 100%O_2_ 2 h	Reduced infarct volume Increasing oxygen supply
Sun	Mice	MCA	25 min	3.0 ATA 100%O_2_ 1 h	Improves energy metabolism Reduce penumbra tissue acidosis
Pushkov	Mice	MCA	30/ 90 min	2.5 ATA 100%O_2_ 1 h	Reduce the infarct volume Improve perfusion
Veltkamp	Rats	MCA	40 min	3.0 ATA 100%O_2_ 1 h	Reduces post-ischemic BBB damage and edema
Kawamura	Rats	MCA	2.5–3.5 h	2.0 ATA 100%O_2_ 0.5 h	Reduced infarct volume Decreased edema
Hu	Rats	MCA	3–6 h	2.0 ATA 100%O_2_ 1 h	Decreased infarction volume Improve neurological function
Badr	Rats	MCA	3/6/12/23 h	3.0 ATA 100%O_2_ 1 h	3/6 h reduced infarct volume 12/23 h increased infarct volume
Yin	Rats	MCA	8 h	2.5 ATA 100%O_2_ 2 h	Reduced cerebral infarction Prevent apoptosis
Lou	Rats	MCA	3/6/12	3.0 ATA 100%O_2_ 1 h	3/6 h protective effects 12 h increased infarct volume

In summary, animal studies have shown that HBOT within 0–12 h of AIS onset and with recanalization can significantly reduce infarct size, stabilize the blood–brain barrier, and improve neurological function. This result raises worries about the clinical applications of HBOT. Some patients with AIS are treated within the time window of this harmful result; it has raised doubts about the value of HBOT in the early application of AIS.

## Clinical exploration of hyperbaric oxygen therapy in patients with acute ischemic stroke

There are three main stages of HBOT for ischemic stroke: pretreatment before onset, early treatment of AIS, and recovery from neurological damage in the chronic phase, of which the most controversial is the application of the acute phase. So, this article focuses on this part. Although the current results of animal studies affirm the value of HBOT in the early application of AIS (within 12 h), there is a mismatch in the translational clinical application of its effective therapeutic window. In addition, the dynamic nature of the ischemic penumbra dictates a relatively short therapeutic window, severely limiting additional treatment application modalities beyond IVT and EVT (40).

The exploration of HBOT for early use in AIS began 20 years ago, with the most far-reaching being the results of clinical trials published in the Stroke Journal in 1991, 1995, and 2003, respectively (Table 2). Two of the three major clinical trials yielded negative results that HBOT was ineffective, and only one trial indicated a possible improvement in outcomes. Rusyniak et al. showed that although HBOT appeared feasible and safe, it did not appear beneficial and harmful in patients with acute ischemic stroke and indicated that they would not continue in more extensive clinical trials (41).

**Table 2:** Randomized controlled trials of HBOT in AIS.

**Authors**	**Patients**	**Ischemia conditions**	**Time window of HBOT**	**HBOT**	**Control**	**Effect**
Anderson	20 HBOT/19 control	Focal ischemia	0–7 d	100% O_2_ 1.5 ATA (1 h/8–9 times)	Air 1.5 ATA (40 min/10 times)	No significant effect
Nighoghossian	17 HBOT/17 control	MCA	0–24 h	100% O_2_ 1.5 ATA (40 min/10 times)	Air 1.5 ATA (40 min/10 times)	Few complications May improve outcome
Rusyniak	17 HBOT/16 control	–	0–24 h	100% O_2_ 2.5 ATA (1 h/ 1 time)	100% O_2_ 1.14 ATA (1 h/1 time)	Few complications No significant effect

Although these randomized controlled trials (RCTs) were noted to have severe deficiencies in sample size, inclusion criteria, the time window for HBOT, and whether reperfusion was restored, leading to unreliable conclusions, the results were not revised, and the trials were not revalidated (17, 31). Whereas the results of other clinical trials and observational studies conducted since then have shown positive treatment effects, they have likewise been noted to have design flaws and poor study quality, making them much less credible (23). In a systematic review published by Bennett MH, validation from 11 clinical trials showed that the results of the HBOT study were insufficient to provide clear guidelines for practice at this time, but did not rule out the possibility of clinical benefit (42). Due to the lack of high evidence-based grades and clear evidence of treatment efficacy, the 2019 American Heart Association/American Stroke Association (AHA/ASA) guidelines for the management of AIS list HBOT as class III, recommending that it “should be offered only in the context of a clinical trial or to individuals with cerebral air embolism” (7).

There is a significant discrepancy between the results of animal experiments and clinical trials. The lack of high-quality studies to change the perception of HBOT, as well as the inconvenience and excessive concerns about safety, has severely limited its clinical application.

## Perspective of hyperbaric oxygen therapy in acute ischemic stroke clinical applications

### Is hyperbaric oxygen a drug or a gas? It is a crucial question

Finding the appropriate therapeutic dose for AIS is critical. The status of hyperbaric oxygen as a drug and the regulations is uncertain or ambiguous in many countries (43). Some commercial organizations perform “off-label indications,” which are often under the supervision of practitioners not burdened with medical qualifications (44). For commercial purposes, it has been labeled a respiratory gas rather than a drug. Non-standardization of application has a negative impact on HBOT. Based on the results of animal models and clinical trials, hyperbaric oxygen has a dose-time effect in terms of its effects. Even if HBO is a gas, its use as a therapeutic agent should only happen with the oversight required for a drug.

Hyperbaric oxygen therapy is used as a uniform therapy, but there are differences in PO_2_, oxygen exposure time, and possible air braking. The differences in HBOT could influence the outcome: (1) The time window for HBOT. As the AIS treatment slogan states (the “time is brain”), the analysis of salvaging the ischemic penumbra above can help us better understand why the time window is so important. Animal studies show that treatment within 12 h is highly neuroprotective, resulting in the most significant benefit to the cerebral cortex. But, the time restriction was not enforced as part of the protocol in the clinical trials; patients received hyperbaric oxygen therapy for more than 12 h or even longer. This has caused HBOT to be beneficial in animal models for specific periods after stroke, but has failed to prove its efficacy in human studies. Furthermore, while the available studies do not explain the harmful effects of treatment beyond 24 h, they also imply that the same time window problem exists in human studies. This may also be one of the reasons for the differences in clinical outcomes and (2) The dose-time effect of HBOT. According to Henry's law, the amount of gas physically dissolved in a gas–liquid system is proportional to the pressure of the gas in the system (45). The effectiveness of treatment is related to the duration and the pressure of HBO treatment. Notably, longer treatment durations and higher pressures can increase oxygen toxicity (described in the next chapter) and damage nerve function, resulting in harmful outcomes.

### Has the clinical translation of hyperbaric oxygen therapy failed in acute ischemic stroke?

It is too early to deny the effectiveness. If animal experiments cannot inform clinical decisions, then serious doubts may arise about the utility of animal models and the ethics of continuing current animal experimental practices (10). The mismatch between the results of animal experiments and the efficacy of clinical trials reminds us that we should re-examine the applicability for clinical applications.

With the development of prehospital emergency modes and treatment modalities, applying more efficient thrombolytic drugs and mechanical embolization techniques has made it no longer challenging to perform HBOT at an early stage. The equipment and concepts of hyperbaric oxygen need to be updated. The bulky, immobile features of the chamber and the high-pressure, pure oxygen environment significantly limit its clinical application and safety concerns. If the treatment programs with lower pressure and lower oxygen concentration (which may not be strictly called hyperbaric oxygen therapy) are effective, safety is significantly increased, treatment costs are reduced, and clinical applications are more convenient. Originated from this idea and the strong desire for neuroprotective agents, the early application of normobaric hyperoxia (NBO) therapy in AIS has been pioneered with exciting pretrial results (46). Patients were randomized and investigated in endovascular therapy (EVT) and NBO (100%, 10 L/min for 4 h) + EVT group. The results suggest that NBO combined with endovascular therapy appears to be a safe and feasible treatment strategy. Compared with endovascular treatment alone, it can significantly reduce infarct volume, improve short-term neurobehavioral test scores, and improve clinical outcomes at 90 days. Compared to the NBO, HBOT has more significant theoretical effects in increased dissolved oxygen and oxygen diffusion radius to achieve salvage of neurological function.

Second, the way of assessing the effect of HBOT needs to be updated. Compared to the traditional clinical function score and the infarct size, functional MRI (fMRI), quantitative electroencephalogram (qEEG), and other responses to neurological function indicators can be used (47). In the study by Cevolani et al., fMRI was first used to evaluate HBOT functional effects on the central nervous system (CNS) of patients with strokes, suggesting a possible role of fMRI in revealing functional neuronal correlates of neuronal plasticity and HBOT-related neoangiogenesis (48).

In addition, the treatment of central retinal artery occlusion (CRAO) provides a reference for cerebral ischemic stroke. CRAO is an ophthalmic emergency presenting as painless, sudden, and severe unilateral vision loss (49). It has a visual progression and is also referred to as a “visualized stroke”. Similar to the principle of salvaging the ischemic penumbra of brain tissue, the oxygen is diffused through the collateral choroidal circulation and the choroid to the inner layers of the retina to reach the salvage of the retina. Patients treated within 12 h of symptom onset showed signs of symptom improvement, with the greatest benefit occurring within 6 to 8 h of treatment (50–52). So, for the future application of HBOT in AIS, specify the optimal therapeutic standard dose (oxygen concentration, treatment window, pressure, and duration of treatment), and improve the accessibility of HBOT in clinical applications, we are very optimistic that the role can be changed from the bench to a starter.

## Adverse effects and safety evaluation of hyperbaric oxygen therapy

Although HBOT has been widely used in a clinical study for more than 50 years, most people have expressed concern about its safety. Consideration of safety is natural, but the excessive concern is completely unnecessary. HBOT is routinely used in clinical practice for <3 ATA and is generally considered safe (23). Under standard treatment, few adverse events have been reported in the clinical use of HBOT in ischemic stroke, with most occurring with ear discomfort (17, 23, 31). Common adverse reactions include central nervous system toxicity, pulmonary and middle ear pneumatic trauma, and, rarely, retinopathy, visual refractive abnormalities, and claustrophobia (53).

Middle ear barotrauma is the most common complication of HBOT, mainly occurring during the first treatment and resulting from the individual's inability to equalize the pressure gradient between the middle ear and the external environment. The main symptoms include ear discomfort, ear pain, ear fullness, and tinnitus and are usually relieved after active manipulation (e.g., Valsalva maneuver) to balance middle ear pressure (54, 55). This discomfort is particularly pronounced in normal or awake patients, while in comatose patients, muscle relaxation greatly reduces this symptom.

Oxygen toxicity is the most severe complication of HBOT and includes pulmonary oxygen toxicity and neurological toxicity. However, there is a dose-concentration relationship for oxygen toxicity, and oxygen toxicity from HBOT is related to the pressure and duration of treatment. Pulmonary oxygen toxicity is mainly caused by the chemical action of oxygen on the respiratory tract and is mainly induced by prolonged exposure to HBOT. Studies have reported significant histopathological changes in 1 ATA for 36 h and 2 ATA for 6 h (56). CNS toxicity is caused by an increase in oxygen radicals and denaturation of neurotransmitters in the CNS and is induced in a pressure- and duration-dependent manner, with studies showing that long- (60–90 min) and high-pressure exposure (6 ATA) induces neuronal cell death. Moreover, oxygen-induced seizures are usually self-limiting and do not require medication to terminate seizure activity. The occurrence of an oxygen-induced seizure can be prevented by shortening the duration of oxygen exposure (inserting more air breaks) or by decreasing the oxygen pressure (57).

## Conclusion

Although the neuroprotective effects of HBOT have been satisfactory in animal models of AIS, it does not match the effect of clinical application. The non-recommended use of AIS management guidelines and the lack of clear indications have made many physicians turn this away. Second, the short treatment window time, within 12 h of onset, the non-portability of HBOT, and safety concerns significantly hamper the application. With the standardization and maturity of AIS treatment, the evidence-based basis should be updated, and it is too early to draw a definite conclusion on the ineffectiveness of HBOT. An effective treatment model of intravenous and endovascular therapy combined with HBOT will give a new meaning to the neuroprotective effect.

## Author contributions

Study concept and design: YY and YH. Data acquisition: YY, BL, and XZ. Manuscript drafting: YY and WF. Review and editing: XA, HR, and YH. Supervision: YH. All authors contributed to the article and approved the submitted version.

## Conflict of interest

The authors declare that the research was conducted in the absence of any commercial or financial relationships that could be construed as a potential conflict of interest.

## Publisher's note

All claims expressed in this article are solely those of the authors and do not necessarily represent those of their affiliated organizations, or those of the publisher, the editors and the reviewers. Any product that may be evaluated in this article, or claim that may be made by its manufacturer, is not guaranteed or endorsed by the publisher.
